# Repair of Adult Mammalian Heart After Damages by Oral Intake of Gu Ben Pei Yuan San

**DOI:** 10.3389/fphys.2019.00607

**Published:** 2019-05-22

**Authors:** Baiping Cui, Yufan Zheng, Xinyan Zhou, Jiaqi Zhu, Jiexian Zhuang, Qianqian Liang, Chen Xu, Wei Sheng, Guoying Huang, Lina Luan, Ning Sun

**Affiliations:** ^1^Department of Physiology and Pathophysiology, School of Basic Medical Sciences–Institute of Integrative Medicine, Fudan University, Shanghai, China; ^2^Jiangsu Vocational College of Medicine, Yancheng, China; ^3^Shanghai Key Laboratory of Birth Defects, Children’s Hospital of Fudan University, Shanghai, China; ^4^Department of Ultrasound, Shanghai Pudong New Area People’s Hospital, Shanghai, China

**Keywords:** heart repair, myocardial infarction, apical resection, traditional Chinese medicine, primary myocardial cells

## Abstract

Adult mammalian heart repair after myocardial damage is highly inefficient due to the post-mitotic nature of cardiomyocytes. Interestingly, in traditional Chinese medicine (TCM), there are reported effective treatments of myocardial infarction (MI) and heart failure in adult humans by oral intake of a TCM concoction named Gu Ben Pei Yuan San (GBPYS), which is composed of *Panax ginseng*, velvet antler, *Gekko gecko* Linnaeus tail, human placenta, Trogopterus dung, *Panax notoginseng*, and amber. We fed mice with GBPYS after myocardial damages through everyday self-feeding. We then examined the effect of everyday oral intake of GBPYS on improving cardiac function and myocardial repair in adult mice after apical resection or MI. We found that long-term oral intake of GBPYS significantly improved cardiac function after myocardial damages in adult mice. BrdU, phospho-histone 3, and AuroraB staining indicated increased cell proliferation at the border zone of MI after TCM feeding. GBPYS feeding reduced organ inflammation, induced angiogenesis, and is non-toxic to mice after long-term oral intake. Further, serum derived from TCM-fed MI rats promoted division of both neonatal rat cardiomyocytes and human induced pluripotent stem cell (iPSC)-derived cardiomyocytes *in vitro*. Oral intake of GBPYS improved heart repair after myocardial damages in adult mice. Our results suggest that there are substances present in GBPYS that help improve adult mammalian heart repair after MI. Also, it could be a good choice of non-invasive alternative therapy for myocardial damages and heart failure after rigorous clinical study in the future.

## Introduction

It is well known that hearts of adult zebra fish, amphibians, or fetal mice within 7 days of birth are capable of complete regeneration after myocardial damages (Poss et al., 2002; Kikuchi et al., 2010; Porrello et al., 2011). In contrast, repair of the adult mammalian heart after myocardial damage is highly inefficient, leading to scar formation and progressive cardiac dysfunction (Leone et al., 2015). It has been reported that, under hypoxia conditions or by manipulating the Hippo–Yapsignaling pathway, heart regeneration in adult mice was induced, and cardiac function after myocardial infarction (MI) was improved (Leach et al., 2017; Nakada et al., 2017). A very recent study reported a combination of cell cycle regulators that induce robust cytokinesis in adult post-mitotic cells and significantly improve cardiac function after myocardial damage (Mohamed et al., 2018). miR-708 also regulates cardiomyocyte proliferation and stress resistance of cardiomyocytes in rodents (Deng et al., 2017). Another study reported that agrin, a component of the neonatal heart extracellular matrix, is required for the full regenerative capacity of neonatal mouse hearts and induced cardiac regeneration in adult mice after MI (Bassat et al., 2017). These latest studies suggest that the poor regenerative capacity of the adult mammalian heart can be enhanced by appropriate exogenous inducers.

In practices of traditional Chinese medicine (TCM), there have been many reports of effective treatments for MI and heart failure using herbs and animal products as medicines. Especially, in the book written by Ke Li (1930–2013), a famous TCM doctor in western China, many local patients with MI were effectively treated by long-term oral intake of his Gu Ben Pei Yuan San (GBPYS) composed of *Panax ginseng*, velvet antler, *Gekko gecko* Linnaeus tail, human placenta, Trogopterus dung, *Panax notoginseng*, and amber (Ke, 2016). In TCM, ginseng is a primary medicine that replenishes *qi* and helps improve regenerative capacity for all kinds of wounds. Velvet antler regenerates every year from the sika deer and thus has very strong regenerative capacity in nature, and it is often used to improve male sexuality. *G. gecko* Linnaeus tails are the regenerative part of the animal and have long been used as a corroborant for weak patients. Human placenta contains many cytokines and substances related to embryonic development and has long been recognized for its beneficial effects for the immune system and improving regenerative capacity after wounding. Trogopterus dung is feces of flying squirrels and is effective in reducing blood stasis. *P. notoginseng* and amber both are used in GBPYS as agents to remove blood stasis. Therefore, the purpose of this concoction composed by Ke Li was to induce regeneration after wounding (infarction is considered as a wound on the heart) and remove blood stasis to improve microcirculation and blood supply during wound healing (Ke, 2016). This concoction comprised equal grams of powder of these seven components, mixed together completely, and was delivered to patients by oral intake of 2–3 g of powder mixture each time and two to three times per day. The treatment effect of GBPYS for local MI patients without any other intervention was significant as reported in Ke Li’s book (Ke, 2016). Most patients were observed with improved heart function, and several patients even showed markedly improved heart repair from MI by echocardiography after 6 months of oral intake of this concoction. These reports suggest that repair could be induced in the adult heart by long-term oral intake of this TCM concoction.

To test the above hypothesis and the effect on myocardial repair in the adult heart of this TCM concoction by oral intake only, we generated both mouse heart apical resection and MI models in the current study and fed them with regular food mixed with this GBPYS concoction powder. It is very interesting that significantly enhanced cardiac cell proliferation was observed within 1-week feeding of this concoction. Transcriptome analyses by RNA sequencing also showed expression changes of many genes regulating cell cycles. We further observed that, after 2 months of GBPYS feeding, heart functions were improved significantly, and the damage size and fibrosis were markedly reduced. Long-term feeding of this concoction not only was non-toxic to mice but also reduced organ inflammation. Although the effective ingredients in this concoction are unknown, it is worth further analyzing in the future to discover new agents enhancing adult heart repair. Taken together, our study showed that adult mammalian heart repair induced by oral intake of TCM is feasible. There are substances improving heart repair present in this TCM concoction that deserves further study. It could be a good choice of non-invasive alternative therapy of myocardial damages in adult patients to use this TCM concoction after further strict clinical studies in the future.

## Materials and Methods

### Traditional Chinese Medicines and Making the Mouse GBPYS Feedstuff

Panax ginsengs were from 16-year-old ginseng roots under forests in Jilin province in China. Velvet antler came from the antlers of sika deer in Jilin province in China. Human placenta, Trogopterus dung, *P. notoginseng*, *G. gecko* Linnaeus tails, and amber were obtained from Anhui Jingquan TCM Decoction Co., Ltd.

The *Chinese Pharmacopoeia* is the code guaranteeing the quality of TCMs, and the microscopic identification section within the *Chinese Pharmacopoeia* sets the quality criterion of each traditional medicine. Microscopic identification of each powdered component of GBPYS showed that all met their set standard quality published in the *Chinese Pharmacopoeia*. All the medicines were completely powdered and mixed well with the regular food for mice at the weight of GBPYS powder vs. regular food at a 5%:95% (low dose) or 10%:90% (high dose) ratio. This ratio was calculated according to the everyday intake amount by the patients described in the book written by Ke Li, the average grams of everyday food intake of mice, and the unit per body surface area [surface area of a person (m^2^) = 0.0061^∗^height (cm) + 0.0128^∗^weight (kg) - 0.1529; surface area of a mouse (m^2^) = 9.1^∗^weight (g)ˆ^2/3^/10,000].

### Animals

All the protocols in this study were approved by the Guide for the Care and Use of Laboratory Animals (National Institutes of Health, Publication No. 85-23, Revised) and were carried out under the supervision of the Fudan University Institutional Animal Care and Use Committee. All experiments were performed on age-matched male mice. C57BL/6J mice and Sprague-Dawley (SD) rats were obtained from Shanghai Slake Laboratory Animal Co., Ltd. R26R-tdTomato and α-MHC-MerCreMer mice were obtained from Shanghai South Model Biotechnology Co., Ltd.

### Mouse Myocardial Infarction Model

Induction of anterior wall MI in mice was carried out using previously described procedures (Wang et al., 2016). Briefly, mice were anesthetized in an airtight chamber using 5% isoflurane, endotracheally intubated, and ventilated using a volume-controlled ventilator with 100% O_2_, supplemented with 2.5% vaporized isoflurane. After lateral thoracotomy and pericardiectomy, the LAD coronary artery was identified along the anterior wall of the left ventricle. Using a 7-0 prolene suture, the LAD artery was ligated at the proximal portion of the artery, close to its origin with the left main coronary artery. Occlusion of the LAD artery resulted in immediate blanching of the anterior wall of the left ventricle, indicative of myocardial ischemia. Subsequently, the chest and the skin were closed in layers using 6-0 silk sutures. Subsequently, the mouse was extubated and then warmed for several minutes until recovery. Feed was immediately changed to feedstuff containing TCM after surgery. At day 3, mice with left ventricular ejection fraction (LVEF) < 40% were selected for subsequent experiments. Mouse hearts were collected at day 7, 1 month, and 2 months after MI.

### Apical Resection

Eight- to nine-week-old mice had surgical removal of the outmost apical region of the heart. Mice were anesthetized with 5% isoflurane and were artificially ventilated with 2.5% isoflurane for maintaining anesthesia after tracheal intubation. Following skin incision, lateral thoracotomy at the third intercostal space was performed by blunt dissection of the intercostal muscles. Following resection, thoracic wall and skin incisions were sutured with 6.0 non-absorbable silk sutures. Mice were then warmed for several minutes until recovery. Feed was immediately changed to feedstuff containing TCM after surgery. At day 3, mice with LVEF < 40% were selected for subsequent experiments. Echocardiographic analysis was performed on day 7, 1 month, and 2 months after the surgery. Hearts were harvested at day 7 after surgery, sectioned, and subjected to immunohistochemistry. For HE or Masson’s trichrome staining, hearts were harvested at 2 months.

### Echocardiography

Assessment of *in vivo* cardiac function on mice was performed with the Vevo 2100 micro-ultrasound system (VisualSonics) at baseline at 3 days post-injury. Echocardiographic M-mode images were obtained from a parasternal short axis view. Echocardiography performed 3 days post-MI demonstrated a severe reduction in LVEF. Echocardiography was then performed 7 days, 1 month, and 2 months after the treatment. LVEF, left ventricular fractional shortening (LVFS), left ventricular diastolic inner diameter (LVIDd), and left ventricular systolic inner diameter (LVIDs) were calculated at all time points.

### 5-Bromo-2-Deoxyuridine Administration

5-Bromo-2-deoxyuridine (BrdU; Sigma) was dissolved in double-distilled H_2_O and filtered. To record cell proliferation *in vivo*, 50 mg/kg BrdU (Sigma) was injected intraperitoneally daily and for 5 days following MI. Hearts were harvested at day 8 and subjected to BrdU IHC using an anti-BrdU antibody (Abcam, #ab152095).

### Lineage Tracing

To track the cell lineage of the newly regenerated cardiomyocytes after MI or apical resection, we intercrossed Myh6-Cre mice with ROSA26-tdTomato mice. Myh6-Cre mice carry the Cre coding sequence inserted after the alpha myosin heavy chain promoter (Myh6), which can drive high-efficiency gene recombination in cardiomyocytes. ROSA26-tdTomato indicator mice harbor a conditional red fluorescent protein variant allele that requires Cre-mediated recombination for expression, and Cre-recombinase is activated by tamoxifen (Sigma), which is dissolved in 90% sesame oil (Sigma) and administered by intraperitoneal injection (1 mg per day per mouse). This system allowed us to clearly visualize RFP-labeled cardiomyocytes *in vivo*. ROSA26-tdTomato and Myh6-Cre mice were maintained on a C57BL/6J background.

### Isolation and Culture of Rat Primary Neonatal Cardiomyocytes

Rat primary neonatal cardiomyocytes were digested from the ventricles of 48-h SD rats using the enzyme mixture of 0.125 trypsin and collagenase II (Invitrogen) in a 37°C water bath, and cultured in Matrigel-coated wells with DMEM/F12 medium supplemented with 15% fetal bovine serum (FBS) at 37°C and 5% CO_2_. In experiments involving administration of 10% serum of MI rats treated with either regular or TCM feedstuff, the cells were allowed to adhere for 72 h prior to treatment. Subsequently, after 24 h of rat serum treatment, regenerative cardiac cells were measured by staining of regeneration markers and contractile force of cardiomyocytes.

### Quantitative Real-Time PCR

Total RNA was extracted using the TRIzol reagent (Life Technologies) according to the manufacturer’s instructions. cDNA was synthesized by the AMV Reverse Transcription System (TOYOBO). Quantitative RT PCR (qPCR) was performed using SYBR Green PCR Master Mix (Bioscience) with GAPDH as an internal control. The nucleotide primer sequences can be found in Supplementary Table S1.

### Histology and Immunofluorescence Staining

Hearts and livers were harvested and fixed in 4% paraformaldehyde (PFA)/PBS solution overnight at room temperature and then processed for either paraffin or cryo-embedding and sectioned. HE and Masson’s trichrome staining were performed according to standard procedures on paraffin sections. The frozen slides were permeabilized with 0.5% Triton X-100 for 15 min and blocked with normal goat serum for 30 min. Then, samples were incubated overnight at 4°C with the following antibodies diluted in 3% BSA blocking solution and 1% goat serum: Anti-cTnT (1:100, MS-295-p, Thermo Scientific). Anti-cTnI (1:200, ab47003, Abcam) antibodies were used to identify cardiomyocytes. Anti-BrdU (1:200, ab152095, Abcam), anti-phosphorylated-histone 3 (pH3) (1:100, 06-570-AF488, Millipore), and anti-AuroraB (1:100, A5102, Sigma) antibodies were used to analyze cell cycle reentry, DNA synthesis, and karyokinesis and cytokinesis, respectively. Other antibodies used in the study included anti-CD31 (1:200, ab28364, Abcam) and anti-actin (1:200, AA132, Beyotime). After three 5-min washes with PBS, samples were stained for 1 h at room temperature with fluorescent secondary antibodies (Abcam) followed by 10 min of DAPI staining for nucleus visualization. Slides were viewed under a fluorescence microscope (Olympus live cell imaging microscope) or spinning-disk confocal microscope (Carl Zeiss). For the quantification of the number of BrdU, pH3, or AuroraB+ cardiomyocytes, the results acquired from at least three to five sections of the heart harvested from each animal at the ventricular valve level of the four-chamber view, or at the level of ligature of the two-chamber view, with at least 100-μm distance from each other were averaged. In all cell counting experiments, fields of view were randomized to reduce counting bias.

### Differentiation of Human Induced Pluripotent Stem Cells and Selection of Pure Ventricular-Like Cardiomyocytes

Differentiation of the human induced pluripotent stem cell (hiPSC) line hiPSC-MYL2^neo/wt^ into cardiac lineage was carried out with the protocol described by Lian et al. (2013). Briefly, hiPSCs were initially cultured in mTeSR1 medium on Matrigel-coated plates until they were ∼90% confluent. Medium was changed to RPMI/B-27 without insulin, consisting of RPMI 1640 (Corning) and B-27 minus insulin (Life Technologies). On day 0–1, medium was supplemented with 12 mol CHIR-99021 (Selleck) in insulin-free RPMI/B-27. After 24 h, IWR-1 (5 mol, Sigma) was added into the fresh RPMI/B-27. On days 5–6, medium was changed to RPMI/B-27 minus insulin. On day 7 of differentiation and every 2 days thereafter, the medium was aspirated, and RPMI/B-27 medium was added. Cultures were maintained in a 37°C, 5% CO_2_ environment. Contracting cells were usually observed from day 8 post differentiation. Selection of pure ventricular-like cardiomyocytes was carried out by adding G418 to the culture as previously described (Li et al., 2017).

### RNA Sequencing

RNA was extracted from fresh tissues using the TRIzol reagent (Life Technologies), and the cDNA libraries were prepared using NGS Multiplex Oligos for Illumina (xCell BioTech Co., Ltd.) according to manufacturer’s instruction. The mRNA was separated from the total RNA and then fragmented and reversed to cDNA, which was end-repaired, 3′ ends adenylated, and barcoded with multiplex adapters. PCR amplified libraries were purified with AmpureXP beads, and samples were quantified by Qubit (Invitrogen) and then run on an Illumina Hiseq (Illumina). Expression abundance estimation and differential expression gene identification were done using OmicsBean, with log_2_ (fold change) > 2 and FDR < 0.05 deemed significant differential expression between the two conditions.

### Fibrotic Scar Area Quantification

Each of the control and GBPYS-fed myocardial infarcted (MI) mouse hearts were paraffin-sectioned and processed for standard Masson’s trichrome staining. The fibrotic area on trichrome-stained sections were quantified with ImageJ (NIH).

### Capillary Density

Tissues were treated with goat serum at 37°C for 30 min and co-immunostaining with anti-CD31 (1:200, ab28364, Abcam) and anti-smooth muscle actin (SMA; 1:200, AA132, Beyotime) at 4°C overnight, followed by a secondary antibody staining. Capillary density was analyzed by ImageJ.

### Blood Routine and Blood Biochemistry Test

Whole blood was collected 3 months after GBPYS administration and subjected to blood routine tests immediately at the Shanghai Laboratory Animal Research Centre (Shanghai, China). Blood was left at room temperature for 1 h and 4°C for 1 h. Following resting, blood was centrifuged at 12,000 rpm for 15 min. The supernatant was used for blood biochemistry tests at the Shanghai Laboratory Animal Research Centre.

### Statistical Analysis

Experimental data were reported as mean ± SEM. To compare the difference between two groups, two-tailed Student’s *t*-test was used. Statistical differences among more than two groups were analyzed with one-way analysis of variance (ANOVA) tests. ^∗^*p* < 0.05 and ^∗∗^*p* < 0.01 were considered statistically significant.

## Results

### Quality Evaluation of the Seven Components of GBPYS and Making Them Into Mouse Feedstuff

**FIGURE 1:**
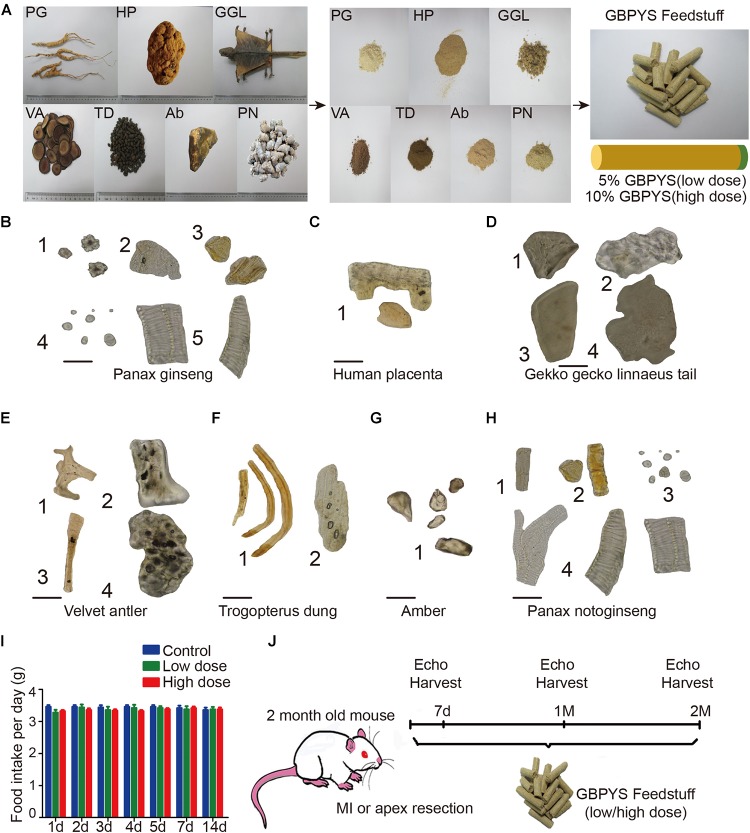
Quality evaluation of the seven components of Gu Ben Pei Yuan San (GBPYS) and diagram of the experimental plan. **(A)** Each component of GBPYS was powdered and mixed well with regular mouse food. Food containing 5% GBPYS powder is defined as low dose and 10% as high dose. PG, *Panax ginseng*; HP, human placenta; GGL, *Gekko gecko* Linnaeus (tail was used); VA, velvet antler; TD, Trogopterus dung; Ab, amber; PN, *Panax notoginseng*. **(B–H)** Microscopic identification of each GBPYS component according to published standards in the *Chinese Pharmacopoeia*. **(B)** The powder of ginseng. (1) Cluster crystal; (2) cork cell; (3) resin canal; (4) starch granules; (5) vessels. **(C)** The powder of human placenta. (1) Fragments. **(D)** The powder of *Gekko gecko* Linnaeus tail. (1) Bone fragments; (2) skin pieces; (3) striated muscle pieces; (4) flake pieces. **(E)** The powder of velvet antler. (1) Cuticula; (2) bone chips; (3) pile; (4) non-ossifying tissue pieces. **(F)** The powder of Trogopterus dung. (1) Fiber; (2) thinner wall cells. **(G)** Amber. (1) Resins. **(H)** The powder of *Panax notoginseng*. (1) Cork cell; (2) resin canal; (3) starch granules; (4) vessels. **(I)** Average everyday diet amount of regular feed, low-dose GBPYS feed, and high-dose GBPYS feed for mice. **(J)** Diagram of experimental plan. Echocardiography was used to detect cardiac function at each time point. MI, myocardial infarction; echo, echocardiography. Scale bars, 50 μm.

To mimic the oral intake process of GBPYS by human patients as in mice, the seven components of GBPYS (Panax Ginseng, human placenta, *G. gecko* Linnaeus tails, velvet antler, Trogopterus dung, amber, and *P. notoginseng*) were all completely powdered and mixed well with the regular mouse feedstuff, with GBPYS taking up 5% (low dose) or 10% (high dose) in total weight of the final food (named the GBPYS feedstuff) (Figure 1A). Quality appraisal by microscopic identification of each powdered component of GBPYS showed that all met their set standard quality published in the *Chinese Pharmacopoeia* (Figure 1B–H). To test the regenerative and therapeutic effect of GBPYS for adult mouse heart after damage, we generated mouse models of apical resection or MI and divided them into five groups: sham surgery mice with control feed, sham surgery mice with high-dose GBPYS feed, disease mice with control feed, disease mice with low-dose GBPYS feed, and disease mice with high-dose GBPYS feed. In the control feed group, mice were fed with regular mouse food every day, while in the other groups, the regular food was replaced by GBPYS feedstuff. Everyday intake amount of the GBPYS feedstuff by the mice was very close to that in the control mice fed with the regular food, at ∼3.2–3.7 g per day (Figure 1I). Mice were all fed for at least 2 months, during which echocardiography was used to evaluate cardiac function at each time point. The whole experimental scheme is shown in Figure 1J.

### GBPYS Feeding Improved Cardiac Function and Reduced Scar Size After Myocardial Damages

**FIGURE 2:**
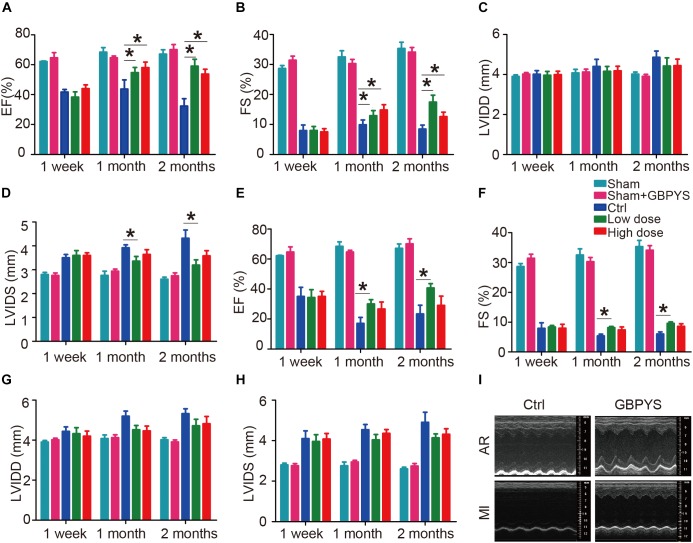
GBPYS feeding improved cardiac function after myocardial damages. **(A–D)** Evaluating treatment effect of GBPYS feeding on apical resection. Compared to control feeding (Ctrl), GBPYS feeding improved cardiac function in mice with apical resection (*n* = 6 each for Ctrl and GBPYS feeding). Left ventricular ejection fraction (LVEF; **A)** and left ventricular fractional shortening (LVFS; **B)** were significantly improved at 1 and 2 months of both low-dose and high-dose GBPYS feeding. Left ventricular systolic inner diameter (LVIDs; **D)** were reduced at 1 and 2 months of low-dose GBPYS feeding. **(E–H)** Evaluating treatment effect of GBPYS feeding on MI. Compared to control feeding, GBPYS feeding improved cardiac function in mice with MI. LVEF **(E)** and LVFS **(F)** were improved in low-dose GBPYS feeding group at 1 and 2 months of feeding. **(I)** Representative echocardiographic images showing improvement in cardiac function after GBPYS feeding in mice with apical resection or MI. One-way ANOVA. ^∗^*P* < 0.05.

Gu Ben Pei Yuan San treatment had no significant effect on cardiac function in the sham group (Figure 2A–H). In mice with apical resection (Figure 2A–D), LVEF (Figure 2A) and LVFS (Figure 2B) improved significantly at 1 and 2 months in both the low-dose and high-dose GBPYS feeding groups compared with those of the control MI mice fed with regular food (*n* = 6, *p* < 0.05). Although LVIDd was not significantly different among groups (Figure 2C), LVIDs was reduced significantly in the low-dose GBPYS feeding group (Figure 2D). It appeared that the low-dose GBPYS feeding resulted in a relatively better effect in reversing the left ventricular function after apical resection at 2 months.

In the mouse MI model (Figure 2E–H), LVEF (Figure 2E), and LVFS (Figure 2F) also improved significantly in the low-dose GBPYS feeding group compared with the control mice fed with regular food at both 1 and 2 months of feeding (*n* = 8, *p* < 0.05). Both LVIDd and LVIDs were reduced in value, although this was not statistically significant (Figure 2G,H). Echocardiographic images showed that GBPYS feeding improved the cardiac function of both MI and apex-resected mice (Figure 2I).

**FIGURE 3:**
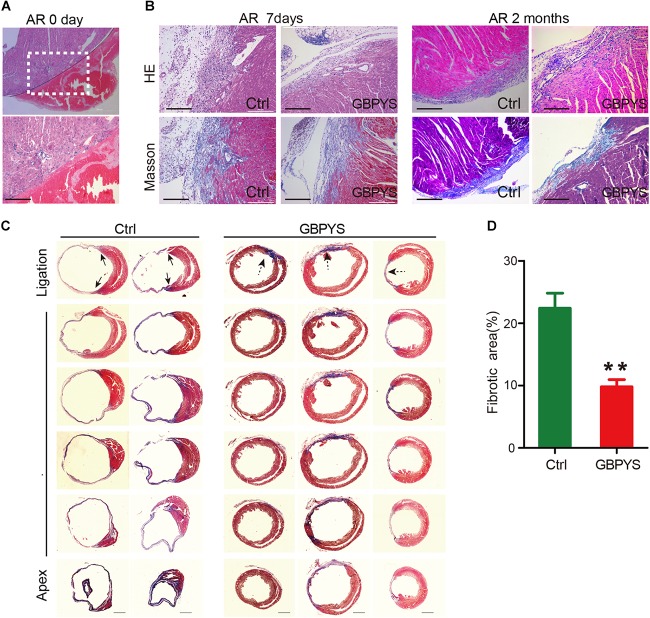
GBPYS feeding reduced scar size after myocardial damages. **(A)** Representative HE staining of heart sections of mice with apical resection at day 0. A hematoma can be clearly observed after apical resection at day 0. The lower panel represents the boxed area in the upper panel. Scale bar, 100 μm. **(B)** HE and Masson’s trichrome staining showed reduced fibrosis area in GBPYS-fed apex-resected mice at 2 months after apical resection. Scale bar, 100 μm. **(C)** Masson’s trichrome staining of serial sections of hearts from MI mice after 2 months feeding, *n* = 8 per group. Solid arrows, scar boundaries; dotted arrows, ischemic region. Scale bar, 2 mm. **(D)** Quantification of fibrotic area relative to myocardium in trichrome-staining sections demonstrated a significant decrease in scar formation in the GBPYS-feeding mice (*n* = 7 for Ctrl and *n* = 8 for GBPYS feeding group). ^∗∗^*P* < 0.01.

Moreover, fibrotic scarring was reduced in GBPYS-feeding mice with apical resection at 2 months (Figure 3A,B). Masson’s trichrome staining of serial heart sections of mice fed with TCM feedstuff 2 months after MI also showed significantly reduced fibrotic area (Figure 3C,D). These data indicate that long-term GBPYS feeding improved heart function and reduced scar size after myocardial damages in adult mice. Because low-dose GBPYS feeding showed a better effect in reversing cardiac function after both MI and apical resection, we used this dose of TCM feeding (5% TCM in regular food) in all subsequent studies.

### GBPYS Feeding Induced Cardiomyocyte Proliferation in Adult Mice With Myocardial Damages

**FIGURE 4:**
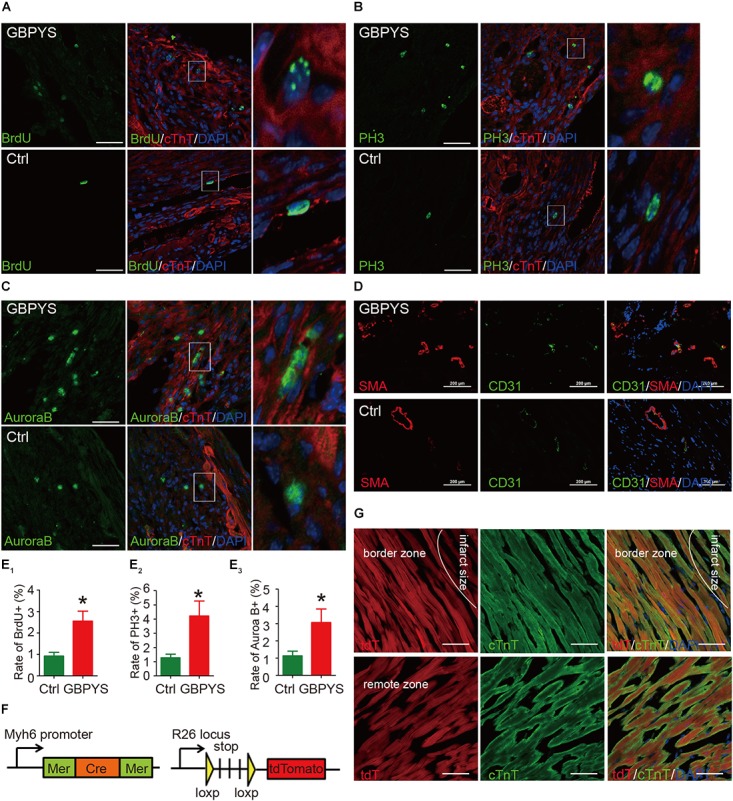
GBPYS feeding induced cardiac proliferation and angiogenesis. **(A,B)** A significant increase in BrdU (green) incorporation was found in cardiomyocytes from GBPYS-feeding MI mice (*n* = 6 each). The right image represents the white boxed area in the upper middle image. Scale bars, 100 μm. ^∗^*P* < 0.05. **(C,D)** Co-immunostaining with anti-PH3 (green) and anti-cTnT (red) antibodies of heart tissue sections from MI mice fed with Ctrl or GBPYS feedstuff showed a significant increase in mitosis in cardiomyocytes from GBPYS-fed MI mice (*n* = 5 for Ctrl, *n* = 6 for GBPYS). The right image represents the white boxed area in the upper middle image. Scale bar, 100 μm. ^∗^*P* < 0.05. **(C–E)** Co-immunostaining with anti-AuroraB (green) and anti-cTnT (red) antibodies showed a significant increase in cytokinesis in cardiomyocytes from GBPYS-fed MI mice (*n* = 5 for Ctrl, *n* = 6 for GBPYS). The right image represents the white boxed area in the upper middle image. Scale bar, 50 μm. ^∗^*P* < 0.05. **(D)** Co-immunostaining with anti-CD31 (green) and anti-smooth muscle actin (red) of heart tissues from MI mice fed with Ctrl or GBPYS feedstuff (*n* = 3 each). Scale bars, 100 μm. **(E)** Quantification of ACMs expressing proliferation marker BrdU, PH3, and AuroraB. **(F,G)** Lineage tracing studies indicated that no newly generated cardiomyocytes were tdTomato-positive. **(F)** A genetic mouse model of tamoxifen-dependent and cardiomyocyte-specific irreversible labeling with fluorescent protein tdTomato. **(G)** Immunostaining of cardiomyocytes (cTnT) and tdTomato in the border zone and remote zone of hearts from GBPYS-feeding mice with MI. Scale bars, 50 μm.

Since GBPYS feeding improved cardiac function and reduced damage size, we next examined whether GBPYS feeding induced regeneration in adult mice after myocardial damage. A mouse MI model was used for subsequent studies as it is most related to human diseases. Heart sections from MI mice were double-immunostained with the cardiomyocyte-specific marker cTnT and one among the cell proliferation marker BrdU, the mitosis marker phosphorylated histone H3 Ser10 (PH3), or the cytokinesis marker Aurora B kinase (AuroraB). We found that, in the GBPYS feeding group, mouse hearts had ∼2.5% cellular BrdU incorporation at 1 week after MI, which was much higher than that in the control mice (Figure 4A,E). We also quantified the number of cTnT-positive cardiomyocytes that were also positive for PH3 staining and found that there was a significant increase in PH3-positive cardiomyocytes in hearts from GBPYS-fed mice compared to mice fed with regular food (Figure 4B,E). Moreover, the number of cells with positive staining of cytokinesis marker AuroraB was significantly increased in GBPYS-feeding mice (Figure 4C,E). Collectively, these results indicated that GBPYS feeding increased cardiomyocyte proliferation and cell cycle reentry in adult mouse hearts at 1 week after MI.

Since the purpose of GBPYS is to promote regeneration (wound healing) and reduce blood stasis after heart damage, we next examined whether GBPYS feeding improved angiogenesis after myocardial damage in adult mice. Heart sections of the border zone of MI were double-stained with the blood vessel marker SMA and CD31. The results showed that GBPYS feeding increased the amount of small blood vessels at the border zone of MI in adult mice fed with GBPYS feedstuff (Figure 4D). Along with the cardiomyocyte proliferation results, this supports an improvement in cardiac function following MI and apical resection by GBPYS feeding.

### Lineage Tracing of the Newly Generated Cardiomyocytes After Traditional Chinese Medicine Feeding in Adult Mice With Myocardial Damage

To determine the lineage origin of those proliferated cardiomyocytes in the hearts of mice fed with GBPYS feedstuff, we utilized the α-MHC-MerCreMer, R26/tdTomato genetic lineage tracing system, where tdTomato specifically and irreversibly labels cardiomyocytes upon tamoxifen induction (Figure 4F). Lineage tracing studies were performed where MI was induced 10 days following tamoxifen administration, and the mice were fed with GBPYS feedstuff immediately after surgery. We found that all of the cardiomyocytes were tdTomato^+^ both in the border zone and in the remote zone (Figure 4G). These results suggest that the newly formed cardiomyocytes were derived from pre-existing cardiomyocytes.

### GBPYS Feeding Induced Global Gene Expression Changes Favoring Cell Proliferation, Angiogenesis, and Repair After Myocardial Damages

**FIGURE 5:**
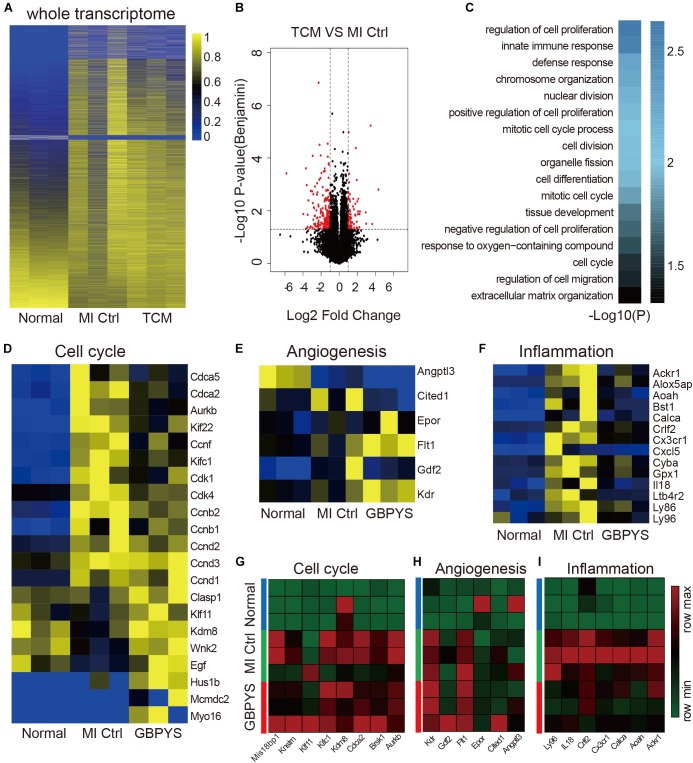
Whole-transcriptomic RNA sequencing showed that GBPYS feeding at day 7 after MI induced complex changes in expression level of cell cycle-, angiogenesis-, and inflammation-related genes. **(A)** Heat map of the whole transcriptomes of all samples indicated that global gene expression after GBPYS treatment is distinct from control feed at day 7 (*n* = 3 per group). **(B)** Volcano plot of the RNA-seq data of GBPYS feeding group versus Ctrl group with regular feeding at day 7 after MI (*n* = 3 per group). **(C)** Gene ontology (GO) analyses of genes with significant change in expression level in the GBPYS feeding group versus the Ctrl group at day 7 after MI (*n* = 3 per group). **(D)** Expression of genes involved in cell cycle at day 7. **(E)** Expression of genes involved in angiogenesis at day 7. **(F)** Expression of genes involved in inflammation at day 7. **(G–I)** Quantitative PCR validation of expression changes of several key genes in cell cycle, angiogenesis, and inflammation pathways from the RNA-seq data at day 7 after MI. *P* < 0.05, Student’s *t*-test.

Since the GBPYS concoction is made up of seven component medicines each containing complex ingredients, we hypothesized that it affected complex multiple molecular pathways to improve heart function and regeneration after damage. To analyze the molecular basis of heart regeneration and functional improvement after GBPYS feeding in mice with myocardial damage, we performed the whole-transcriptome RNA sequencing of the heart tissues from the border zone 1 week after MI. The results showed that, after GBPYS feeding, the global mRNA expression patterns of heart tissues from MI mice were clearly different from those of the control MI mice fed with regular food (Figure 5A). There were a total of 70 genes upregulated and 185 genes downregulated in the heart tissues from the border zone of MI after GBPYS feeding (Figure 5B). Gene ontology analyses revealed that genes involved in mitotic nuclear division, cell cycle, immune response, and chromosome organization were enriched in the GBPYS feeding group (Figure 5C). Cell cycle genes that are key to regulating cardiomyocyte proliferation, such as ccnd1, ccnd3, clasp1, and kdm8, were upregulated (Figure 5D). In addition to genes regulating cell cycle, upregulation of key genes of angiogenesis, such as Flt1 and Kdr (Figure 5F), were also observed in GBPYS-feeding mice. The most significant event was that genes involved in inflammation were downregulated (Figure 5E), suggesting less inflammation in GBPYS-feeding mice. These results support the effect of GBPYS feeding on cell proliferation and cell cycle changes, angiogenesis, and inflammation in the heart tissues from adult MI mice. We further verified the RNA sequencing results by qPCR, which indicated upregulation of certain genes in the cell cycle and angiogenesis as well as downregulation of genes in inflammation (Figure 5G–I). Of note, there was also downregulation of certain genes associated with cell cycle regulation (Figure 5D), indicating that the effect of GBPYS feeding on myocardial damage was a multiplex process.

Further analyses of signaling pathways from the transcriptomic sequencing data of heart tissues after 1 week of GBPYS feeding in MI mice suggested that there were changes associated with tumor and immune disease pathways (Supplementary Figure S1A). We further validated some of the gene expression changes in the main pathways that GBPYS acts on and found that, compared with the control group, genes in p53 and Hippo signaling pathways that inhibit cell cycle, including E2f2, Nupr1, Pmaip1, Sox11, and WWC1, were significantly downregulated in the GBPYS treatment group (Supplementary Figure S1B). These results again suggest that cell proliferation was promoted in the heart tissues of MI mice after GBPYS feeding.

**FIGURE 6:**
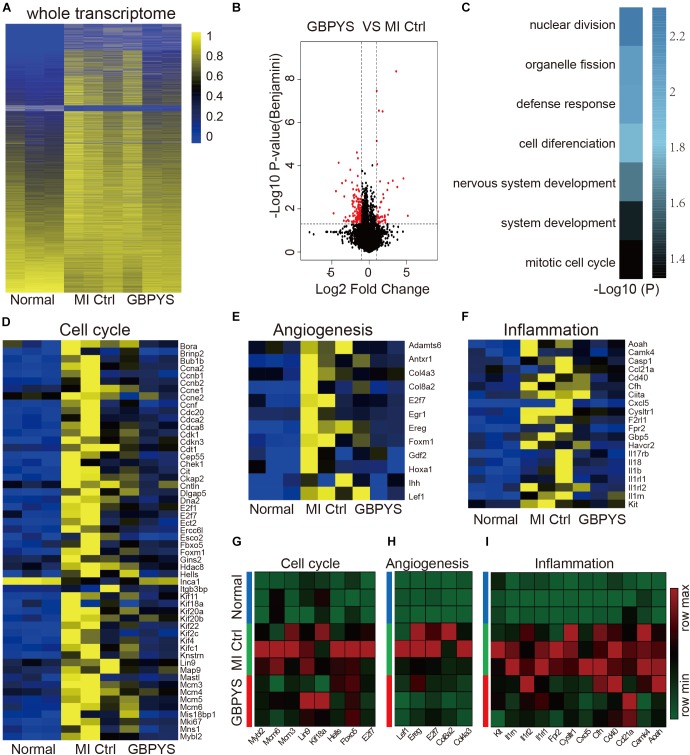
Whole-transcriptomic RNA sequencing showed that gene expression patterns of the GBPYS feeding group at 1 month after MI were more similar to those in normal mice. **(A)** Heat map of the whole transcriptomes showed that, compared with MI Ctrl, the gene expression patterns of GBPYS-feeding mice at 1 month after MI were more similar to those of normal mice (*n* = 3 per group). **(B)** Volcano plot of the RNA-seq data of GBPYS feeding group versus control group with regular feeding at 1 month after MI (*n* = 3 per group). **(C)** GO analyses of genes with significant change in expression level in the GBPYS feeding group versus the Ctrl group at 1 month after MI (*n* = 3 per group). **(D)** Expression of genes involved in cell cycle at 1 month. **(E)** Expression of vessel development genes at 1 month. **(F)** Expression of genes involved in inflammation at 1 month. **(G–I)** Expression changes of several key genes in cell cycle, angiogenesis, and inflammation pathways from the RNA-seq data at 1 month after MI were validated by qPCR. *P* < 0.05, Student’s *t*-test.

To see the effect of prolonged GBPYS feeding on adult mouse heart repair, we further performed whole-transcriptome RNA sequencing of heart tissues from the border zone 1 month after MI (Figure 6A–F). Compared with normal mice without MI and control MI mice fed with regular food, the global expression pattern of the whole transcriptome of GBPYS feeding was closer to that of the normal mice (Figure 6A). Compared with the control MI mice, there were 46 genes upregulated and 133 genes downregulated after GBPYS feeding (Figure 6B). The differentially expressed genes of GBPYS-feeding mice were enriched in the processes of cell–cell adhesion, nuclear division, defense response, etc. (Figure 6C). At this time, expression levels of genes related to cell cycle, angiogenesis, and inflammation in GBPYS-feeding mice were close to those in normal mice without MI and lower than those of the control MI mice (Figure 6D). Some of these genes that exhibited significant change in expression level were further confirmed by qPCR (Figure 6G–I). These results suggest that, at 1 month after MI, a lot of repair events were almost finished in the GBPYS feeding group, and this led to the status of the heart tissues being close to normal.

### GBPYS Reduced Inflammation in Important Organs, and Prolonged GBPYS Feeding Was Not Toxic to Mice

**FIGURE 7:**
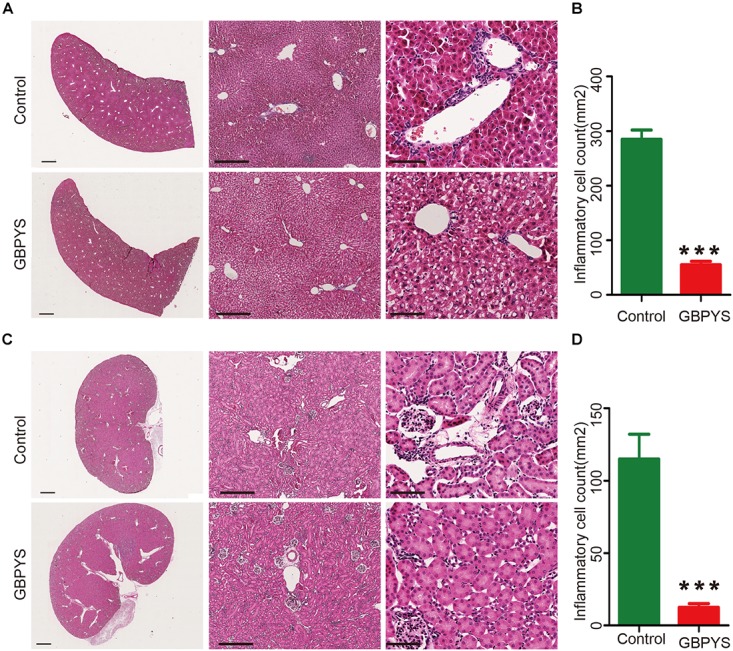
GBPYS feeding for 7 days alleviated inflammation of liver and kidney in mice with MI. **(A)** GBPYS feeding for 7 days reduced the accumulation of inflammatory cells around the hepatic junction in mice with MI compared with Ctrl MI mice fed with regular food (*n* = 6). **(B)** Quantification of inflammatory cells around the hepatic junction from six liver slides (*P* < 0.001). **(C)** GBPYS feeding for 7 days reduced the accumulation of inflammatory cells around the blood vessels of the kidney in mice with MI compared with control MI mice fed with regular food (*n* = 6). **(D)** Quantification of inflammatory cells around the blood vessels from six kidney slides. Scale bars, 2 mm, 200 μm, 50 μm, respectively. ^∗∗∗^*P* < 0.001.

**FIGURE 8:**
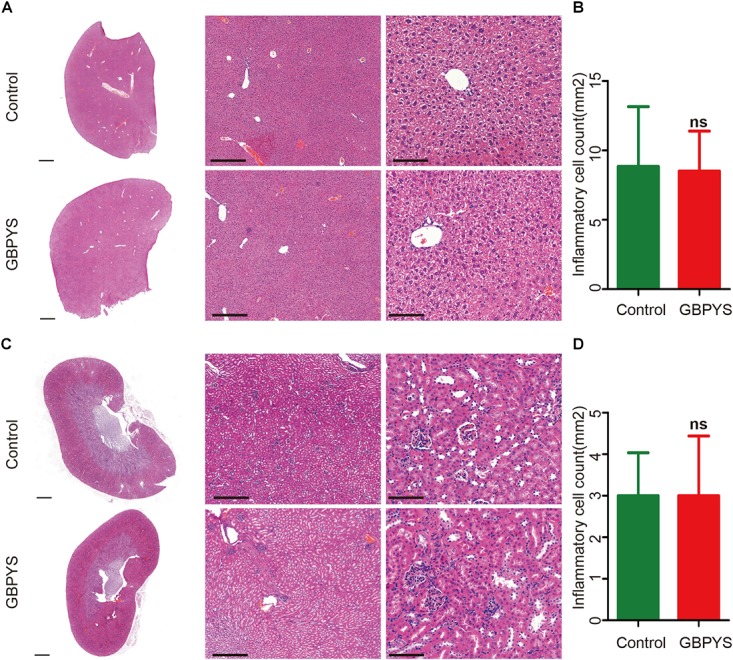
GBPYS feeding for 3 months had no apparent toxicity to liver and kidney in normal mice. **(A)** GBPYS feeding for 3 months had no significant toxicity to liver in normal mice compared with Ctrl normal mice fed with regular food (*n* = 6). **(B)** Quantification of inflammatory cells around the hepatic junction from six liver slides. **(C)** GBPYS feeding for 3 months had no significant toxicity to kidney in normal mice compared with Ctrl normal mice fed with regular food (*n* = 6). **(D)** Quantification of inflammatory cells around the blood vessels from six kidney slides. Scale bars, 2 mm, 200 μm, 50 μm, respectively. ns, *P* > 0.005.

Drugs and toxins were metabolized mainly through the liver and kidney following oral administration. GBPYS required long-term feeding of MI mice, and it may cause acute or chronic toxicity to the liver and kidney. To see whether GBPYS feeding is toxic, we examined the kidney and liver from MI mice with GBPYS feeding at 1 week by HE staining. GBPYS feeding alleviated inflammation of the liver in mice with MI compared with control feed (Figure 7A). Also, around the hepatic junction, liver sections showed a difference in inflammatory cell infiltration between control and GBPYS-fed mice (Figure 7B). One-week GBPYS feeding reduced the accumulation of inflammatory cells around the blood vessels of the kidney in mice with MI when compared with control MI mice fed with regular food (Figure 7C). Quantification of inflammatory cells around the blood vessels in the kidney showed a notable decrease (Figure 7D). To further identify the types of inflammatory cells in liver and kidney tissues, immunostaining with CD68 for macrophages and CD3 for T-cells was performed (Supplementary Figures S2A–D). The results showed that the GBPYS group had less CD68^+^ macrophage infiltration in the liver and kidney tissues compared with the control group (Supplementary Figures S2A,B). No CD3^+^ T-cell infiltration in the liver and kidney in both GBPYS and control groups was found (Supplementary Figures S2C,D). These results were in agreement with our data from whole-transcriptome RNA sequencing showing that many inflammation genes were downregulated at 1 month (Figure 5F,H). To test the toxicity of long-term GBPYS feeding, we analyzed organ structures and inflammation by HE staining in mice fed with GBPYS feedstuff for 3 months (Figure 8A–D) and found that there was no obvious toxicity in liver and kidney tissues. Further, the routine blood test and the blood biochemistry test showed no obvious abnormality after 3 months of GBPYS feeding for mice with MI when compared with MI mice with control feeding (Table 1), suggesting that prolonged GBPYS feeding did not result in significant toxicity in living mice.

**Table 1:** GBPYS feeding for 3 months did not show obvious toxicity in MI mice.

	Control	Treatment	*P*
**Routine blood tests**		
WBC (10ˆ9/L)	3.3 ± 0.4	3.4 ± 0.8	0.905306156
RBC (10ˆ12/L)	9.7 ± 0.3	9.7 ± 0.2	0.843271371
PLT (10ˆ9/L)	15.1 ± 1.2	14.5 ± 1.7	0.753657526
HGB (g/L)	142 ± 3.5	142.5 ± 3	0.91748452
NEUT (10ˆ9/L)	1 ± 0.4	0.9 ± 0.3	0.901837146
LYMPH (10ˆ9/L)	1.4 ± 0.1	1.5 ± 0.1	0.6189209
MONO (10ˆ9/L)	0.1 ± 0	0.1 ± 0.1	0.778082013
EO (10ˆ9/L)	0.1 ± 0	0.1 ± 0.1	0.898364069
BASO (10ˆ9/L)	0	0	NS
**Blood biochemistry test**		
TP (g/L)	48.3 ± 1	47.5 ± 0.6	0.580110235
ALB (g/L)	29.5 ± 0.6	29 ± 0.4	0.536963324
GLOB (g/L)	18 ± 0.4	18.3 ± 0.4	0.704852545
ALT (U/L)	24.8 ± 1.4	23.8 ± 1.7	0.717670992
AST (U/L)	70 ± 4	66.3 ± 4.5	0.610456327
LDH (U/L)	342 ± 25.5	324 ± 26	0.683347429
Urea (mmol/L)	14.5 ± 1	14.3 ± 0.5	0.858802346
CREA (μmol/L)	6.8 ± 0.2	5.3 ± 0.5	0.055084961


### Serum From GBPYS-Feeding Rats Enhanced Proliferation of HiPSC-Derived Cardiomyocytes and Primary Neonatal Cardiomyocytes *in vitro*

**FIGURE 9:**
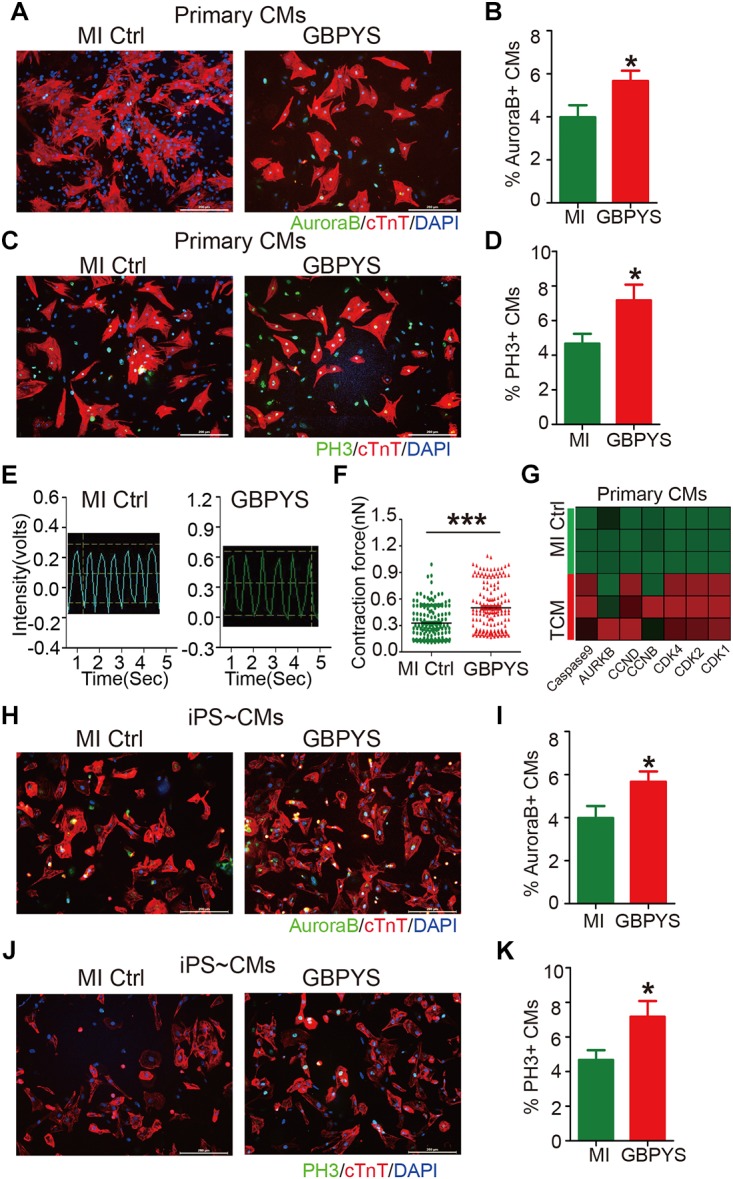
Serum from MI rats fed with GBPYS feedstuff enhanced proliferation of hiPSC-derived cardiomyocytes and neonatal rat cardiomyocytes. **(A–F)** Neonatal rat cardiomyocytes treated with serum from MI rats fed with Ctrl or GBPYS feedstuff. **(A)** Cells were stained with DAPI (blue), cTnT (red), and AuroraB (green). Scale bar, 200 μm. **(B)** Rate of AuroraB-positive cardiomyocytes in response to serum from MI rats fed with Ctrl or GBPYS feedstuff. **(C)** Cells were stained with DAPI (blue), cTnT (red), and PH3 (green). Scale bar, 200 μm. **(D)** Rate of PH3-positive cardiomyocytes in response to serum from MI rats fed with Ctrl or GBPYS feedstuff. Data are mean ± SEM. ^∗^*P* < 0.005. **(E)** Representative traces of contraction force recording of rat neonatal cardiomyocytes treated with serum from MI rats fed with Ctrl or GBPYS feedstuff. **(F)** Quantification of relative contraction force of rat neonatal cardiomyocytes treated with the respective serum. Each dot in the plot represents contractility of a single cardiomyocyte. Data are mean ± SEM. ^∗∗∗^*P* < 0.001. **(G)** Quantitative PCR examinations of expression level of several key cell cycle-related genes after 24 h of serum treatment. *P* < 0.05, Student’s *t*-test. CMs, cardiomyocytes. **(H–K)** HiPSC-derived cardiomyocytes treated with serum from MI rats fed with Ctrl or GBPYS feedstuff. **(H)** Cells were stained with DAPI (blue), cTnT (red), and AuroraB (green). Scale bar, 200 μm. **(I)** Numbers of AuroraB-positive cardiomyocytes in response to serum from MI rats fed with Ctrl or GBPYS feedstuff. Data are mean ± SEM. ^∗^*P* < 0.005. **(J)** Cells were stained with DAPI (blue), cTnT (red), and PH3 (green). Scale bar, 200 μm. **(K)** Rate of PH3-positive cardiomyocytes in response to serum from MI rats fed with Ctrl or GBPYS feedstuff. Data are mean ± SEM. ^∗^*P* < 0.005.

After oral intake, GBPYS must be eventually digested by the digestive system, and all the effective ingredients that induce cell proliferation and heart repair finally enters the blood circulation. Therefore, the serum must contain effective ingredients that induce cardiomyocyte proliferation. To test this hypothesis, we collected the serum from MI rats fed with control regular food or MI rats fed with the GBPYS feedstuff for a week, and added to the culture media maintaining primary neonatal rat cardiomyocytes or hiPSC-derived pure cardiomyocytes *in vitro*. Compared with the serum from MI rats fed with control regular food, serum from GBPYS-feeding MI rats significantly enhanced proliferation of neonatal cardiomyocytes (Figure 9A–D) and hiPSC-derived cardiomyocytes (Figure 9H–K). Also, serum from GBPYS-fed MI rats enhanced contraction force of neonatal cardiomyocytes after 24 h of treatment (Figure 9E,F). We found that serum from GBPYS-fed MI rats significantly increased the expression level of CCND, CCNB, CDK1, CDK2, CASPASE9, AURKB, and CDK4, genes that are key to induce cell cycle reentry and proliferation of cardiomyocytes, in primary neonatal cardiomyocytes (Figure 9G). These results indicated that the effective ingredients inducing cardiomyocyte proliferation were truly present in the serum after oral intake of this TCM concoction.

## Discussion

In this study, we report an interesting phenomenon where oral intake of GBPYS induces repair of the adult mammalian heart after myocardial damages. This TCM concoction is composed of seven components in equal amounts: *P. ginseng*, velvet antler, *G. gecko* Linnaeus tail, human placenta, Trogopterus dung, *P. notoginseng*, and amber. These medicines are all regularly used in TCM practices nowadays in China. The purpose of this concoction designed by the TCM doctor Ke Li was to induce cardiac wound repair after myocardial damage and to improve microcirculation after infarction. Indeed, we observed that GBPYS treatment by oral feeding induced cell proliferation and cell cycle reentry as well as angiogenesis in the hearts of adult mice with apical resection or MI. GBPYS treatment significantly improved cardiac function and reduced fibrosis area after myocardial damage in adult mice. The overall increased level of cell proliferation and cell cycle reentry is comparable to the hypoxia- and agrin-induced level reported by Bassat et al. (2017) and Nakada et al. (2017), respectively, suggesting a comparable effect in inducing adult heart repair using this TCM concoction.

The complexity of this TCM combination and the many ingredients in each component medicine make it difficult for us to analyze the effective ingredients and their mechanisms of action in inducing adult mammalian heart regeneration in this study. Actually, the mechanisms of stem cells or engineered heart tissue transplantation and injectable hydrogel treatment for myocardial damages are also very complex, so that the effective factors involved are still not yet fully determined. Our transcriptome RNA sequencing data showed that expressions of many cell cycle, inflammation, and angiogenesis genes were significantly changed after GBPYS feeding, suggesting that multiple complex pathways were involved in the process of inducing cardiac cell proliferation and heart repair. This complexity may be required for effective treatment for myocardial damage, which is of course a complex disease process. As pointed out by Dr. Joseph Hill in his recent review, manipulating a single pathway may not be efficient in effectively reversing the complex process of pathological cardiac remodeling after myocardial damages (Burchfield et al., 2013; Xie et al., 2013). Almost all clinical studies based on manipulating a single target to treat heart failure or MI thus far have resulted in failure (Kajstura et al., 1997; Sabbah et al., 2000; Dor et al., 2003; Yao et al., 2004; Annex and Simons, 2005; Padwal and Majumdar, 2007). Interestingly, clinical trials of some Chinese herbal formulas showed significant therapeutic effect in patients with chronic heart failure (Wang et al., 2012; Li et al., 2013). In addition, clinical trials have shown that some combinatory therapies effectively treated metabolic diseases (Jonnalagadda et al., 2018; Yu et al., 2018). These examples suggest that effective treatment of complex diseases may require combinatory medicines. The wisdom of TCM, such as using the seven component medicines in Ke Li’s concoction, may give us more suggestions for better designing future clinical treatment and drug development activities for heart diseases. We believe it is worth further investigating into each component medicine for GBPYS in the future to uncover the multiple effective ingredients in inducing heart repair, thereby helping to improve the design of treatment strategies for myocardial damages.

Another important point from our current study is that oral intake of this TCM concoction means that it was digested by the stomach and all cytokines or proteins were eventually denatured and lost their function. However, our data showed that serum from MI rats fed with GBPYS feedstuff promoted cell cycle reentry and proliferation of cardiomyocytes *in vitro*. This suggests that either there are some ingredients beyond proteins that are not destroyed by the stomach acid and act on inducing cardiomyocyte proliferation or there are some factors inducing cardiomyocyte proliferation that were activated by GBPYS feeding and released into the blood circulation. Further studies are worth it to analyze those unknown ingredients that may have the capacity to induce cardiomyocyte proliferation, and these surely will be important discoveries if any are found.

**FIGURE 10:**
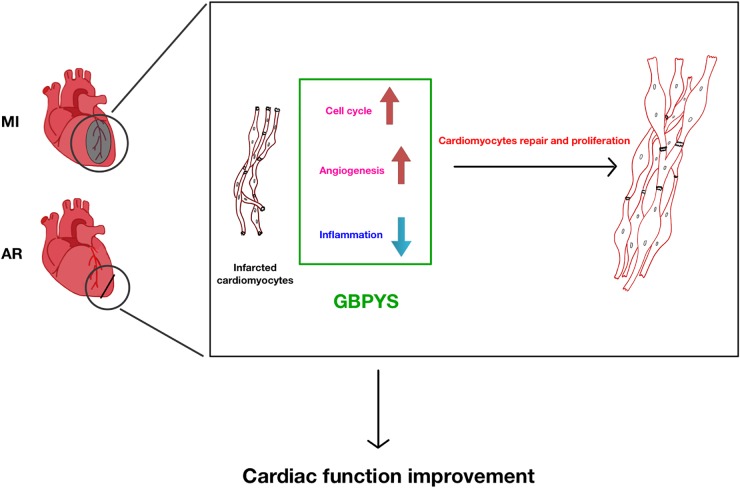
Summary of GBPYS effects on heart after myocardial damage in adult mice. MI, myocardial infarction; AR, apical resection; GBPYS, Gu Ben Pei Yuan San.

Overall, the significance of our current study is that it shows that oral intake of GBPYS helps induce repair of the adult mouse heart after myocardial damages (Figure 10). To our knowledge, there was previously no such report showing that adult cardiomyocyte proliferation can be induced by oral intake of medicines. Furthermore, long-term oral intake of this GBPYS concoction did not show significant toxicity to important organs as reflected by blood routine and blood biochemical tests as well as pathological examinations. Our data supported the observation described in the TCM doctor Ke Li’s book that many local patients with MI improved their cardiac function after receiving GBPYS treatment. Since this GBPYS concoction has been used on many humans already, it is worth further designing strict clinical studies to confirm previous treatment effects in human patients and make this TCM concoction an alternative non-invasive treatment choice for patients with MI in addition to stent implantation. We hope this will bring more benefits for patients with MI or even severe heart failure.

## Ethics Statement

All the protocols in this study were approved by the Guide for the Care and Use of Laboratory Animals (National Institutes of Health, Publication No. 85-23, Revised) and was carried out under the supervision of the Fudan University Institutional Animal Care and Use Committee. All experiments were performed on age-matched male mice. C57BL/6J mice and Sprague-Dawley (SD) rats were obtained from Shanghai Slake Laboratory Animal Co., Ltd. R26R-tdTomato and αMHC-MerCreMer mice were obtained from the Shanghai South Model Biotechnology Co., Ltd.

## Author Contributions

BC, NS, and LL wrote the manuscript. BC, YZ, XZ, JqZ, and JxZ performed the experiments. QL and CX analyzed the data. WS and GH conducted the experiments.

## Conflict of Interest Statement

The authors declare that the research was conducted in the absence of any commercial or financial relationships that could be construed as a potential conflict of interest.

## Supplementary Material

FIGURE S1 The analysis of cardiac tissue signal pathways after 1 week of GBPYS feeding in MI mice. **(A)** Interaction of cardiac tissue pathways after 1 week of GBPYS feeding in MI mice. Circles and stars represent genes, and squares represent related diseases. The size of the graph represents the amount of gene expression. **(B)** Expression changes of several key genes in p53 and Hippo pathways after 1 week of GBPYS feeding in MI mice were validated by qPCR. ^∗^*P* < 0.05, ^∗∗∗^*P* < 0.001, Student’s *t*-test.

FIGURE S2 GBPYS feeding for 7 days reduced the macrophage infiltration of the liver and kidney in mice with MI. **(A)** GBPYS feeding for 7 days reduced the CD68^+^ (red) macrophagocyte around the central veins in mice with MI compared with Ctrl MI mice fed with regular food (*n* = 6). **(B)** GBPYS feeding for 7 days reduced the CD68^+^ (red) macrophagocyte around the blood vessels of the kidney in mice with MI compared with control MI mice fed with regular food (*n* = 6). There is no CD3^+^ (green) T-cell infiltration of the liver **(C)** and kidney **(D)** in both GBPYS and control groups. Scale bars, 50 μm.

TABLE S1 The nucleotide primer sequences of qPCR used in Figure 6, 7.
