# Supplementary figures and images for: Repair of Adult Mammalian Heart After Damages by Oral Intake of Gu Ben Pei Yuan San
Source: Front Physiol. 2019 May 22;10:607. doi: 10.3389/fphys.2019.00607 (PMC6541202; doi:10.3389/fphys.2019.00607)

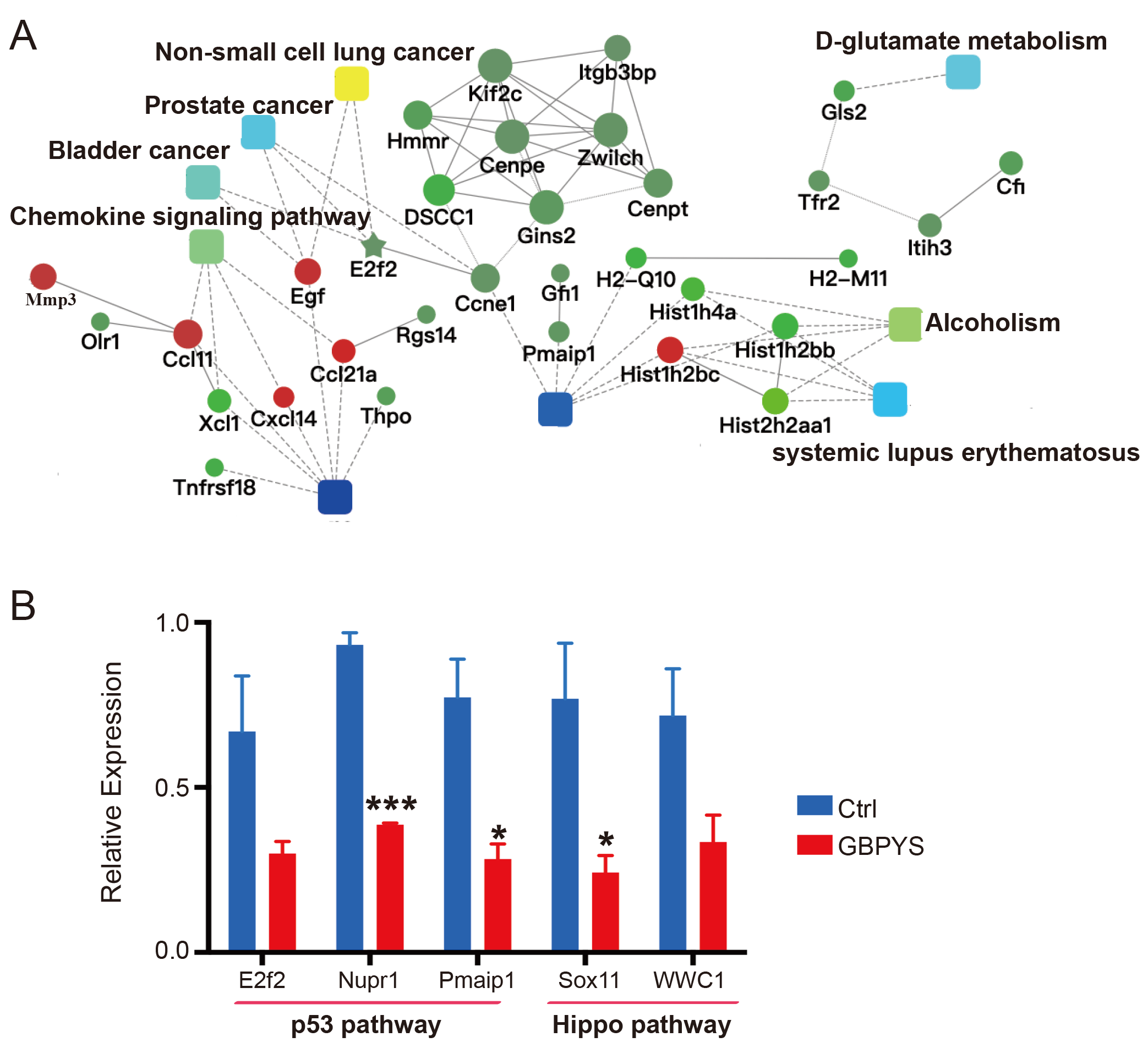

Supplement: FIGURE S1 — The analysis of cardiac tissue signal pathways after 1 week of GBPYS feeding in MI mice. (A) Interaction of cardiac tissue pathways after 1 week of GBPYS feeding in MI mice. Circles and stars represent genes, and squares represent related diseases. The size of the graph represents the amount of gene expression. (B) Expression changes of several key genes in p53 and Hippo pathways after 1 week of GBPYS feeding in MI mice were validated by qPCR. ∗P < 0.05, ∗∗∗P < 0.001, Student’s t-test. [file Image_1.TIF]

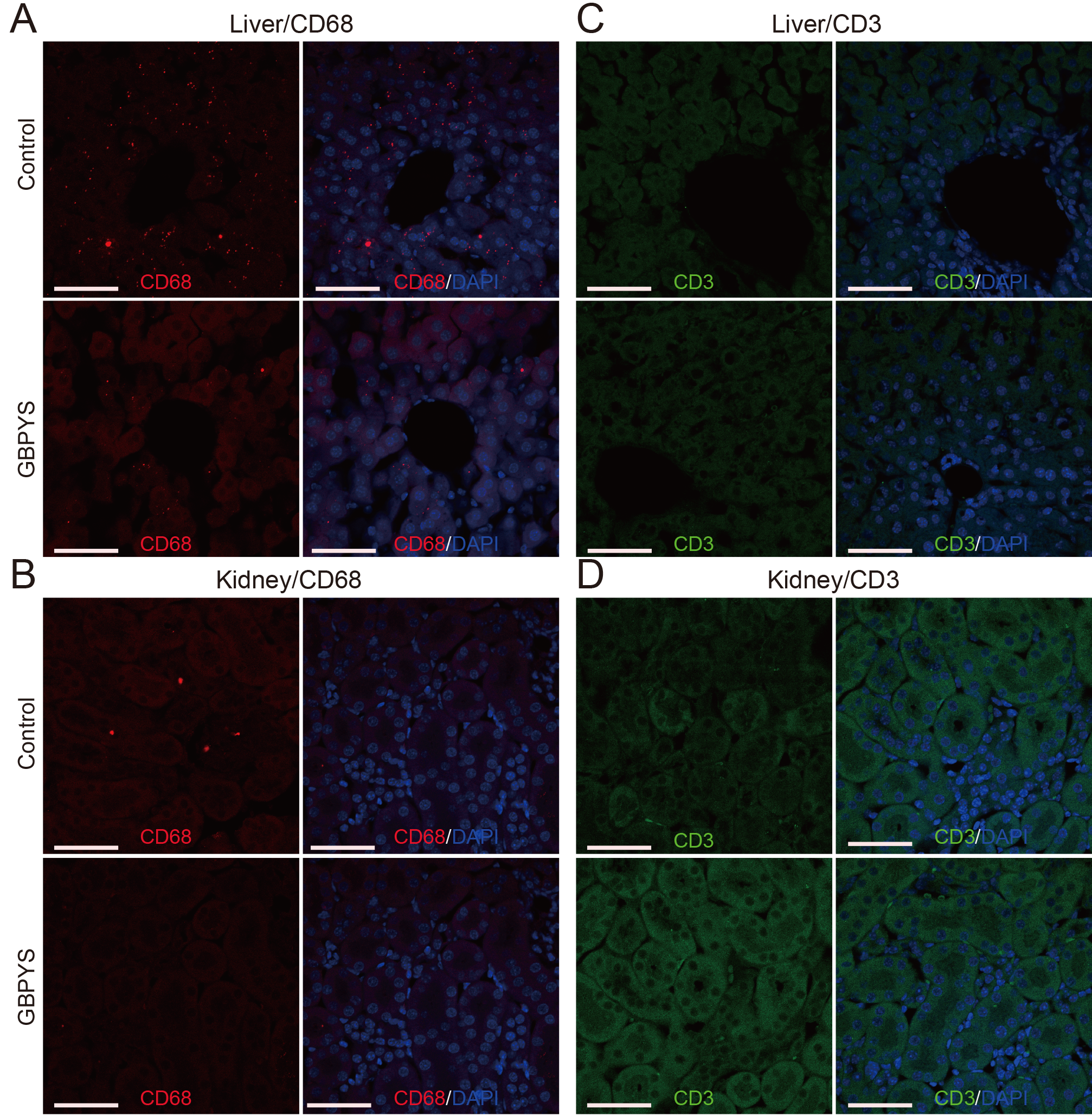

Supplement: FIGURE S2 — GBPYS feeding for 7 days reduced the macrophage infiltration of the liver and kidney in mice with MI. (A) GBPYS feeding for 7 days reduced the CD68+ (red) macrophagocyte around the central veins in mice with MI compared with Ctrl MI mice fed with regular food (n = 6). (B) GBPYS feeding for 7 days reduced the CD68+ (red) macrophagocyte around the blood vessels of the kidney in mice with MI compared with control MI mice fed with regular food (n = 6). There is no CD3+ (green) T-cell infiltration of the liver (C) and kidney (D) in both GBPYS and control groups. Scale bars, 50 μm. [file Image_2.TIF]
