# Supplementary material for: Repair of Adult Mammalian Heart After Damages by Oral Intake of Gu Ben Pei Yuan San
Source: Front Physiol. 2019 May 22;10:607. doi: 10.3389/fphys.2019.00607 (PMC6541202; doi:10.3389/fphys.2019.00607)
Supplement: TABLE S1 — The nucleotide primer sequences of qPCR used in Figure 6, 7. [file Table_1.DOCX]

**TABLE 1. The nucleotide primer sequences of qPCR used in figure 6 and figure 7.**

7 day primers

| Primers | forword | reverse |
| --- | --- | --- |
| Ackr1 | CCTGGGCTACTGGGTATGGT | AGGCGCAGAAGCCATTGTAA |
| Angptl3 | ACAGCCCTTCAACACAAGGT | GGGGAGTAGTTCTTGGTGCT |
| Aoah | GCTCAATCCCTGTAACGGCT | CCCAGACACGATTTGCTTGC |
| Aurkb | GCACCTGAAACATCCCAACAT | ACCTAACAGCAGGTTCTCCG |
| Brsk1 | TTGAGCACGTTTCTGGTGGT | CAGGGACGCCATACCAAAGT |
| Calca | TCTCCCCTTTCCTGGTTGTCAG | CAGTGACACTAGAGCCCTCAGC |
| Cdca2 | TCACACGACAAGCCTCTCTC | ATTGGTTTCTGGAGAGCCCC |
| Cited1 | CCTTGGAGTGAAGGATCGCA | GCCCCTTGGTACTGGCTATT |
| Crlf2 | TGCGATGGCTCTTCTGGAAC | GACAGGAAATATCGCGGGCA |
| Cx3cr1 | AATCTGTTGGTGGTCCTCGC | CTGCACTGTCCGGTTGTTCA |
| Epor | CCCCTCTGTCTCCTACTTGC | GGCAGTGAACACCAGAAACG |
| Flt1 | CGACCCTCTTTTGGCTCCTT | ATCTTCACAGCCACAGTCCG |
| Gdf2 | AGTATCGAGTGCCGTGAAGC | CTGCTCATGGCCGATCATCT |
| Il18 | GTAAGAGGACTGGCTGTGACC | GGCAAGCAAGAAAGTGTCCTTC |
| Kdm8 | TGTCAGAGGACACCACAGAGC | GGACCAGTGTTGAGCTTTTCC |
| Kdr | CAGGCAACATCGGTCCACAT | TGGTCTCGCCAATGGTTGTT |
| Kifc1 | AGGCCACCTTTGTTGGAAGT | ACCAATAGCAGAGCACCCAC |
| Klf11 | AGACGGACATCGAAGCTGTG | CTTGGGAAGGAACAGGGGTC |
| Knstrn | AGCAAAGGTCTCAACCCAGG | GTCACTGGCTTCGTCCTTACA |
| Ly96 | ATTCCAAAGTTGCCGAAGCG | CGGCGGTGAATGATGGTGAA |
| Mis18bp1 | GGTCAGATGTGGCTATGGCA | TGCAAGGGTGTTGCAGTGAA |

1 month primers

| Primers | forword | reverse |
| --- | --- | --- |
| Camk4 | ACGGGGTGCTACATCCATTG | GCTCAAGGACCAGGCTGATT |
| Ccl21a | ATCCTGTTCTCACCCCGGAA | TTGAGGGCTGTGTCTGTTCA |
| Cd40 | GCTATGGGGCTGCTTGTTGA | AGTGTCTGTGCTGGTGACAG |
| Cfh | AGGACAGGAGAACAAGTGACAT | CACATCACTTCCACTTGCCCA |
| Col4a3 | GAGAACCATCCGTAGGCAGG | CATTCAAGCCGGGAAGTCCT |
| Col8a2 | CGTGGGAGGGGTCTACTACT | GCCTGATCTGAGGGCATCTG |
| Cxcl5 | GTTCCATCTCGCCATTCATGC | CTATGACTTCCACCGTAGGGC |
| Cysltr1 | AGCCAGGTTCGTTTGCATTG | GCCTTCCTACGACTTGGCAT |
| E2f7 | GCCCACAGGGAGAAAGACAA | ACACCTACTTGTCCGCTTGG |
| Ereg | GGTCTTGACGCTGCTTTGTC | CCAGTGTAGCCCACTTCACA |
| Fbxo5 | CAAGAAAATGGGCCTGGAGC | CCCAGGTCGATGACTTTTGAAC |
| Fpr2 | TGCTATGGACTCATTGCTGTCA | TGCTATGGACTCATTGCTGTCA |
| Hells | GGGACTTGAGAAAGAGCGGA | GGTTTCTCTCCATTCCCATCA |
| Il1rl1 | GGTTGCTCTGTTCTGGAGAGAT | ATCATGTGAGGGGCCAGAAC |
| Il1rl2 | AAGACTACAGCACCATGCCC | CCTGTGGCAGCATTGCATTT |
| Il1rn | GGGACCCTACAGTCACCTAAT | TGGATGCCCAAGAACACACTA |
| Kif18a | GTCACCATTTGCACCTCCAG | GCTTTGGTTGTTCGGCAGTT |
| Kit | TGACGGTACATGGCTGCATT | GATAGTCAGCGTCTCCTGGC |
| Lef1 | ACGCTAAAGGAGAGTGCAGC | AGCTTCTCTTACCACCTGAAGT |
| Lin9 | AGTTTCTGGCTCTGACACGG | CTGGTCTGGTGCAAGCTCAT |
| Mcm3 | ATGACCAGCAAGATACCCGC | TCCAGTGTCCGTGCTGTAAC |
| Mcm6 | GTACAAACGTCTCCGCCAGA | GTACAAACGTCTCCGCCAGA |
| Mybl2 | CAAGCGACAGAAGAAACGGC | GGGAGTACTTCTGATGATGGATAC |
